# Design and Engineering of Natural Cellulose Fiber-Based Biomaterials with Eucalyptus Essential Oil Retention to Replace Non-Biodegradable Delivery Systems

**DOI:** 10.3390/polym14173621

**Published:** 2022-09-01

**Authors:** Flávia P. Morais, Joana M. R. Curto

**Affiliations:** 1 Fiber Materials and Environmental Technologies (FibEnTech-UBI), Universidade da Beira Interior, R. Marquês de D’Ávila e Bolama, 6201-001 Covilhã, Portugal; 2 Chemical Process Engineering and Forest Products Research Centre (CIEPQPF), Universidade de Coimbra, R. Sílvio Lima, Polo II, 3004-531 Coimbra, Portugal

**Keywords:** 3D modeling and simulation, carboxymethylcellulose, eucalyptus essential oil, microfibrillated cellulose, natural cellulose fibers, sustainable biomaterials

## Abstract

This work aims at the design and engineering of sustainable biomaterials based on natural fibers to replace non-renewable fiber sources in the development of non-woven delivery systems. Cellulose fibers were used as the main support to produce multi-structured materials with the incorporation of microfibrillated cellulose (MFC) as an additive. A 3D carboxymethylcellulose matrix retaining a natural bioactive product, eucalyptus essential oil, (CMC/EO), with controlled release functionalities, was also applied to these materials using bulk and spray coating methodologies. Additionally, using a 3D modeling and simulation strategy, different interest scenarios were predicted to design new formulations with improved functional properties. Overall, the results showed that MFC provided up to 5% improved strength (+48%) at the expense of reduced softness (−10%) and absorbency (−13%) and presented a good potential to be used as an additive to maximize natural eucalyptus fibers content in formulations. The addition of CMC/EO into formulations’ bulk revealed better strength properties (21–28%), while its surface coating improved absorption (23–25%). This indicated that both application methods can be used in structures proposed for different sustainable applications or a more localized therapy, respectively. This optimization methodology consists of a competitive benefit to produce high-quality functionalized biomaterials for added-value applications.

## 1. Introduction

Over time, interest in the development of new natural fiber materials has increased to replace non-biodegradable systems. This fact is due to cellulosic sources presenting advantages as renewable, sustainable, and economical resources [1]. The new era of low basis weight and high porosity materials, such as non-woven delivery systems for therapeutic applications, aims to increase the quality of products for personal use due to their multiplicity, profitability, cleanliness, and convenience. The latest and newest trend in these applications is facial masks. These traditional masks have been reformulated over the years with new aspects, ingredients, vehicles, and perspectives for several purposes [2,3]. These single-use products present softness, strength, and a good absorption and penetration profile, providing rapid and deep hydration [2,4]. Many of these products are composed of non-renewable resources, such as synthetic fibers or plastic derivatives, which present serious environmental impact issues [5,6].

Cellulose-based biomaterials have been presented as one of the solutions to sustainability problems, as well as a biocompatible, biodegradable, and versatile system to deliver and release active substances like natural ingredients from plant extracts [7,8]. It is important to find simple and environmentally friendly solutions and strategies to manufacture natural fiber-based delivery systems for sustained and controlled release of essential oils to be applied over a long period of time [9,10,11]. Recently, essential oils have been proposed to present activity against the SARS-CoV-2 virus, as they present anti-inflammatory, immunomodulatory, bronchodilatory, and antiviral properties [12]. da Silva et al. (2020), [13], also hypothesized that essential oil components may interact with key COVID-19 protein targets. For this purpose, a molecular docking analysis was performed using different essential oil components with different proteins from SARS-CoV-2. The results indicated the docking energies were relatively weak, meaning they are unlikely to interact with the virus targets. However, essential oil components can act synergistically, potentiate other antiviral agents, or provide some relief of COVID-19 symptoms [13]. An example of these natural products is eucalyptus essential oil (EO), which has been used as an antioxidant, anti-inflammatory, antispasmodic, decongestant, deodorant, disinfectant, antiseptic, antibacterial, insecticide, stimulant, and healing agent, among other features [14,15,16,17,18]. These applications depend on the potential of each eucalyptus species [19,20,21].

Biomaterial development processes influence the quality, performance, efficiency, and economic value of the products. According to previous works, modified cellulosic materials can have a high positive impact on functional properties [22]. The production of modified pulps at the microscale level proves to be an economical alternative, with its specific use in these applications [23,24]. Microfibers are identified as a highly suitable material for self-care products, as they increase hydrogen bonds which, in turn, increase inter-fiber bonds and structural strength [25]. Microfibrillated cellulose (MFC) fills empty spaces and increases inter-fiber bonds. Selective densification of the structures can be achieved using MFC and producing multilayer biomaterials [26]. The potential to combine the different functional properties with microfiber properties could enhance the production of products with multilayer functionalities. The design and development of a multi-structured material can contribute to an innovative strategy for new applications [27]. An example that can be highlighted is the preparation of a multilayer product produced with facial paper materials and non-woven face masks for personal protection associated with the SARS-CoV-2 disease [28,29]. This strategy allows absorption of the water vapor exhaled from the mouth and nose and reduces the compression of the skin on the nose [28]. The biomaterials can be manufactured to achieve a certain thickness, bulk, and water vapor absorption capacity. The design and development of products with optimized structural and absorption properties can allow the replacement of plastic-based synthetic filters used in individual protection masks, being a sustainable and affordable alternative. The addition of cellulose-based alternatives to polypropylene in non-woven materials can significantly improve their performance in the prevention of respiratory infections, for example [30,31].

Another challenge in biomaterials functionalization is the use of optimized 3D networks that function as a vehicle to controllably retain and release active molecules. Carboxymethylcellulose (CMC), due to its characteristics of low toxicity, biocompatibility, hydrophilicity, and biodegradability, has been widely used in these advanced delivery systems applications [32,33]. A CMC structure forms a strong 3D polymeric network capable of holding water or biological substances, presenting a sustained release with prolonged and located action in a specific area of the body, with lesser adverse side effects [34]. The development of new materials requires innovative methodologies to produce structures with desired functionalities. The modification of these materials can be carried out by physical and chemical crosslinking, or even the direct immersion of structures with additive solutions for absorption and dissolution [35,36]. In addition, two other methods included the addition into the bulk of the prepared formulations to produce the structures and the application of layer suspension coating. The last technique is normally established by spraying to develop materials, in which the solution of interest is directly deposited on the surface of structures [36,37,38]. To the best of our knowledge, the application in bulk and by the spray of a CMC formulation with therapeutic molecules, such as eucalyptus essential oil, in cellulose-based structures has not been widely investigated in the literature.

**Figure 1:**
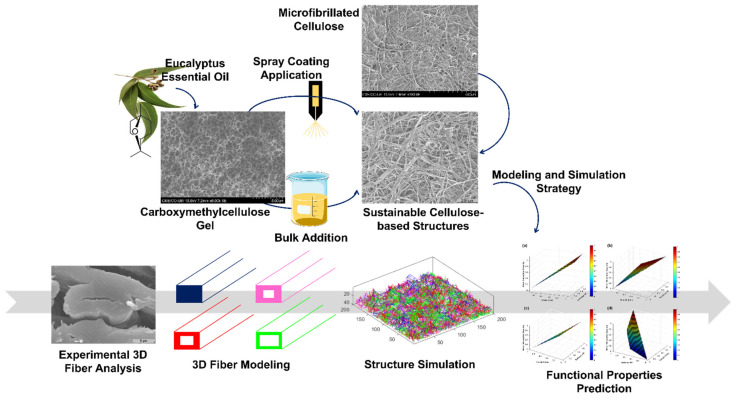
Experimental and computational design plan to carry out the present work [33].

In this context, the main goal of this work was to develop, engineer, and design natural fiber-based structures with innovative features aimed at added-value non-woven delivery system applications. Figure 1 presents the experimental and computational design plan to carry out this work. Structures based on cellulose wood fibers were prepared with the incorporation of MFC at different percentages, and CMC/EO structure. The CMC was used as a vehicle to retain and transport the EO. Both the effect of MFC as an additive and the effect of the CMC/EO added to the bulk and applied by spray were investigated. For this purpose, morphological, chemical, and water interaction characteristics of raw materials and formulations were analyzed. Low basis weight and high porosity structures were produced, and the softness, strength, and absorption properties were measured and predicted using a 3D fiber and structure modeling and simulation approach, and artificial intelligence tools such as interpolation and extrapolation models, multiple linear regression, and artificial neural networks.

## 2. Materials and Methods

### 2.1. Materials

An industrial never-dried, total chlorine-free (TCF) bleached Eucalyptus kraft pulp (hardwood) was used in this study, with a Shöpper-Riegler degree (°SR) of 25°. Additionally, an industrial air-dried, elemental chlorine-free (ECF) bleached Pinus kraft pulp (softwood) was subject to a mechanical treatment of refining using a lab-scale PFI-mill beater to reach the same target of 25 °SR, according to ISO 5264/2. CMC sodium salt with high viscosity (1500–3000 cps) in 1% H_2_O (25 °C), an average molecular weight of 700,000 g/mol, and a degree of substitution of 0.65–0.90 (carboxymethyl group per anhydroglucose unit) was purchased from Sigma Aldrich (Darmstadt, Germany) and used in this study as an additive. Eucalyptus essential oil (EO) was purchased from Medical Plants Department store, physalis^®^. To produce the MFC sample, an industrial air-dried, ECF bleached Eucalyptus kraft pulp was used.

### 2.2. Production and Characterization of MFC

ECF bleached Eucalyptus kraft pulp was hydrated in a basic aqueous solution (pH 12) and disintegrated following ISO 5263, at 30,000 revolutions. The cellulose fiber suspension was mechanically treated at 9000 revolutions and 3.33 N/mm refining intensity, according to the ISO 5264/2 standard PFI mill at 10% (*w*/*w*). After this treatment, the fibers were treated with 1 mg of enzyme/g of pulp for 180 min, under conditions of pH 7, consistency of 4% at 40 °C, with continuous mechanical agitation, using a propeller impeller to ensure the efficacy of mass transfer, as described by [39].

The morphological properties of MFC fibers were evaluated automatically by a system based on image analysis, the MorFi Analyzer (TECHPAP, Grenoble, France) of a diluted suspension of 20 mg/L in a flow chamber. The studied parameters included the number of fibers, fiber length weighted by length, fiber width, coarseness, kinked fibers, curl, rate of macrofibrils, and fines elements content [40]. The results were determined in triplicate. Additionally, the scanning electron microscopy (SEM) method was also used to characterize the MFC structure, using a Hitachi S3400 N-II model (Tokyo, Japan), with 20 kV accelerating voltage at different magnifications. An MFC laboratory-made structure was prepared to perform this analysis, being initially coated with gold using a Sputter Quorum Q 15 OR ES (Laughton, East Sussex, UK). The method for preparing laboratory structures is shown below.

The sample drainability measured by °SR method was evaluated in triplicate according to ISO 5267/1.

The water retention value (WRV) was accessed by MFC sample centrifugation, in triplicate, according to the method reported by [41]. Two grams of the fiber suspension was centrifuged with a centrifugal force of 3000× *g* for 10 min. Posteriorly, the samples were removed, weighed, and oven-dried at 105 ± 2 °C for 24 or more hours. After this procedure, the samples were again weighed. The WRV index (%) was calculated as *WRV = ((M*_1_ − *M*_2_*)/M*_2_*) ×* 100, where “*M*_1_” corresponds to the mass of the wet sample after centrifugation and “*M*_2_” corresponds to the mass of the dried sample after the oven.

The intrinsic viscosity, [ƞ], was measured according to the ISO 5351 by dissolving the samples in cupriethylenediamine (CED) solution. The degree of polymerization (DP) was calculated from viscosity using the Mark–Houwink–Sakurada equation (DP = [ƞ]/0.57) as reported by [42].

The carboxyl group content (C_COOH_) was determined according to the TAPPI T 237 om-93 standard, in which samples were acidified with 0.1 M HCl, filtered, mixed with NaHCO_3_/NaCl solution, and titrated with 0.01 M HCl. The millimoles of acid groups per 100 g of pulp were accessed by the HCl volume spent in the titrations, the mass of water retained in the sample after filtration, and the mass of dried mass.

Fourier-transform infrared spectroscopy with attenuated total reflectance (FTIR-ATR, Thermo-Nicolet IS10, Waltham, MA, USA) was performed with a resolution of 4 cm^−1^, at a wavelength range of 600 to 4000 cm^−1^. A MFC laboratory-made structure was used to perform this analysis.

### 2.3. Production and Characterization of CMC/EO Structure

The CMC/EO structure was prepared with CMC solution at 5 mg/mL (0.5% (*m*/*v*)), under controlled temperature (25–35 °C), agitation (500 rpm), and pH (6.5–9) conditions, and about 3 mL (1800 mg) of eucalyptus essential oil was added to this suspension. The durability of the eucalyptus essential oil-based fragrance was monitored over time.

SEM method was used to characterize the CMC structure. In a first instance, to maintain the porous structure, the CMC sample was immersed in a 2.5% (*m*/*v*) glutaraldehyde solution for 24 h. Subsequently, the sample was treated with ethanol solutions of graduated concentrations (10 min each) to replace the water with ethanol. Then, the sample was dried by the CO_2_ Critical Point Drying method (EMS K850 Critical Point Drier) with thermo-electronic heating, adiabatic cooling, and temperature control of +5 °C in cooling and +35 °C in heating [32]. This method consists of using CO_2_ to replace any water in the sample by a series of dehydration. The samples are placed in the pressure chamber, which is pre-cooled and immediately filled with CO_2_ liquid from the gas cylinder, with a critical point at 31 °C and 1072 psi. It heats up just above the critical temperature, reaching a critical pressure, under working conditions around 35 °C and 1500 psi. After this treatment, the sample was coated with gold using a Sputter Quorum Q 15 OR ES (Quorum Technologies, Lewes, UK). Finally, the samples were analyzed using a Hitachi S3400 N-II model (Tokyo, Japan), with 20 kV accelerating voltage at different magnifications.

The CMC/EO structure was evaluated by rheological analyses using a stress-controlled rheometer (RheoStress^®^ RS 150, Haake Technik GmbH, Vreden, Germany), at room temperature of 23 ± 1 °C. In both flow and oscillation modes, a cone and plate geometry with a 2° angle cone sensor (C35-2°), a diameter of 35 mm, and a gap of 0.105 mm were used. In the flow mode, controlled rate flow tests were performed with a shear rate (ẏ) in the range of 0.03 to 1000 s^−1^ for 180 s to determine the shear stress (T) and viscosity (ƞ). In the oscillation mode, viscoelastic behavior was studied with shear stress in the range of 0.07 to 1000 Pa and a frequency of 0.01 Hz to analyze the storage modulus (G′) and loss modulus (G″) [43]. The frequency value was selected by measuring G′ and G″ parameters at frequencies ranging from 0.01 to 1.0 Hz and applying a chosen stress value, allowing measurements within the linear viscoelastic region. The results were performed in duplicate.

The components of EO were identified by a Gas Chromatography-Mass Spectrometry (GC-MS), using an Agilent Technologies 7890A GC A.01.13 equipped with an Agilent Technologies 5975 inert XL MSD along with Triple-Axis Detector.

FTIR-ATR method was also used to identify the chemical functional groups, as described above. A dried CMC sample was used in the assays.

The differential scanning calorimetry (DSC) method (Netzsch, DSC-204, Selb, Bavaria) was performed with a temperature range of 18 to 150 °C and a velocity of 2 °C/min, in order to investigate the stabilization, retention, and release of structures with CMC/EO structure. About 6 to 18 mg of sample were used, and its mass before and after the assays was quantified.

### 2.4. Preparation and Characterization of Formulations

Table 1 presents the formulations used in this study. Formulation F1 represents a benchmark consisting of 75% eucalyptus fibers and 25% softwood fibers. Formulation F2 was carried out to investigate the effect of MFC as an additive in this industrial reference. In formulations F3 and F4, the CMC/EO structure was applied in the bulk of the two formulations mentioned, while in formulations F5 and F6, it was applied by spraying on the surface of the dry laboratory-made structures. Formulations F7 to F9 were performed to investigate the effect of reducing softwood pulps in order to maximize eucalyptus pulps with the MFC combination. These three formulations were further compared with other mixtures in the same range of our previous study [24]. All formulations were also characterized in terms of morphological properties using MorFi analyzer, pulp drainability measured by °SR method, and WRV. These methods were described above.

### 2.5. Preparation and Testing of Laboratory-Made Structures

The laboratory-made structures were prepared with the formulations described in Table 1, according to an adaptation of ISO 5269/1. This adaptation included the suppression of the pressing operation and the production of structures with a basis weight of 20 g/m^2^ instead of 60 g/m^2^, to mimic the low-basis weight materials. The samples were also conditioned at (23 ± 1) °C, with a relative humidity of (50 ± 2)%, as described in ISO 187 standard. 

The morphology of the structures’ 3D network was accessed by SEM, as described above, using a Hitachi S-2700 model (Tokyo, Japan).

The structural properties of the structures, such as basis weight, thickness, and bulk were determined following ISO 12625-6 and 12525-3 standards. Thickness was measured using a FRANK-TPI^®^ micrometer (ISO 12525-3); the basis weight was calculated through the quotient between the average mass of each structure and the respective area of 0.02138m^2^ (ISO 12625-6), and the bulk was calculated through the quotient between the thickness and the basis weight (ISO 12525-3). The apparent porosity was calculated as *Porosity = 1 − (structure density/cellulose density of 1.5 g/cm^3^)*.

The functional properties of softness, strength, and absorption were predicted using a computational simulation approach, as described below. More detailed information about the experimental validation of these data can be found in our previous publication [44]. Briefly, these methods included tensile index (ISO 12625-4), water absorption capacity (ISO 12625-8 adaptation), capillary rise (ISO 8787 adaptation), and softness using a Tissue Softness Analyzer (TSA Emtec), with QAI algorithm to determine the handfeel (HF) and TS7 parameters. The standard adaptation to determine the water absorption capacity consists of immersing 2.5 g of sample in a basket for 30 s. After this period, the basket is removed from the water, running on the support, with an amplitude of 30° for 60 s. Subsequently, the sample was weighed to determine the amount of water absorbed by the structures. The assay was performed in triplicate. The standard adaptation to determine capillary rise consists of collecting the water rise in the 50 mm wide sample at 10, 20, 30, 60, 180, 300, and 600 s. Four samples were used to perform the tests. The assays were performed in a conditioned room at a temperature of (23 ± 1) °C and a relative humidity of (50 ± 2)%, according to ISO 187.

### 2.6. Computational Studies and Statistics

**Figure 2:**
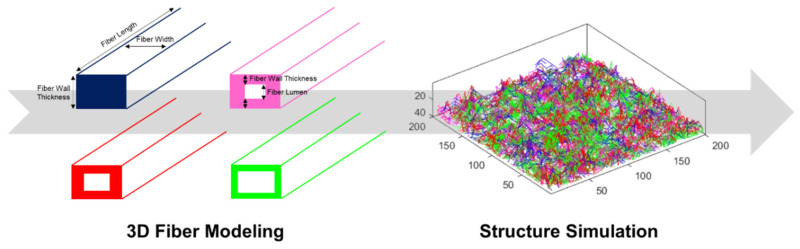
Representative scheme of 3D modeling and computational simulation approach, in which different fibers are represented with different colors. The fibers are modeled according to their dimensions and properties, considering the three fiber dimensions. Modeling is detailed to the point where fiber wall thickness, fiber lumen, flexibility, and degree of collapse are considered. The models consist of fibers without lumen and with different lumen dimensions [46]. Finally, 3D structures are simulated from these proposed models in order to predict structural properties and, consequently, optimize functional properties.

A 3D modeling and simulation strategy was used to predict the functional properties, which considers the structural hierarchy at the fiber and structure level. To accomplish these studies, a 3D simulator of fibrous materials, named *voxelfiber*, was used [45,46], available on https://github.com/eduardotrincaoconceicao/voxelfiber (accessed on 1 June 2022). Briefly, the fibers were modeled in 3D from their classification into different categories, according to fiber wall thickness, degree of collapse, and fiber flexibility. A 3D structure made from these modeled fibers was obtained. Figure 2 presents a scheme of this 3D modeling and computational simulation approach. The prediction and establishment of relationships between the functional properties were achieved through the programming of calculation engine algorithms and database integration, computational tools for 3D modeling, and mathematical models, such as interpolation and extrapolation models, multiple linear regression, and artificial neural networks. More detailed information about this computational methodology can be found in our previous publications [44,45,46,47]. In this work, different scenarios of the formulations with fiber mixtures and additives incorporation were compared and predicted. The variables were normalized to present the same scale range, and computational studies were also carried out using MATLAB^®^ (R 2020a, 9.8.0.1323502, MathWorks, Natick, MA, USA). Statistical analysis was performed using the IBM SPSS Statistics 25 (Armonk, NY, USA), and R statistical software version 3.6.3.

## 3. Results and Discussion

### 3.1. Characterization of MFC

**Figure 3:**
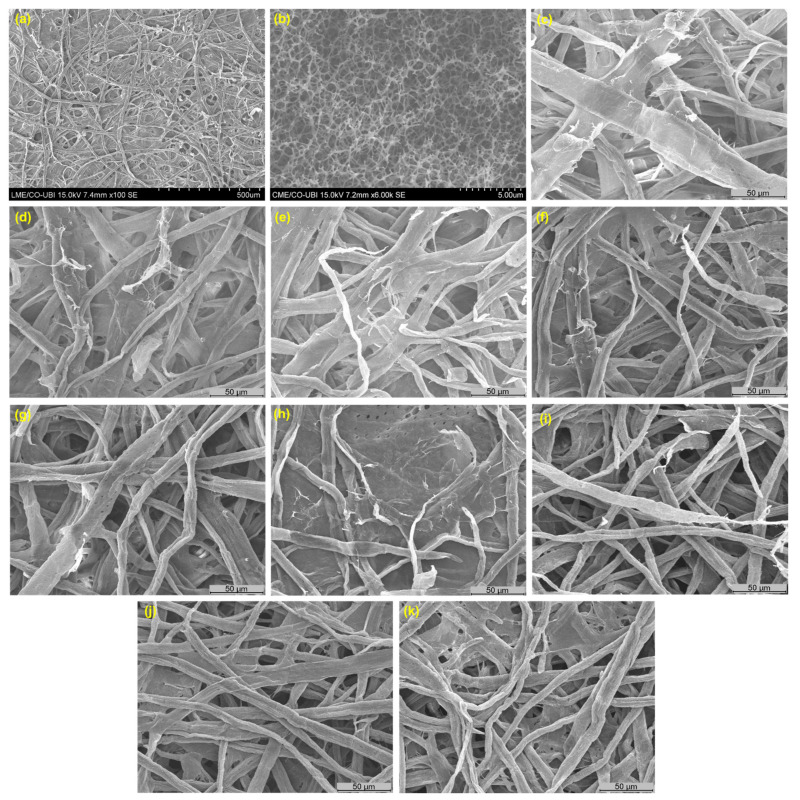
SEM image of morphology and 3D network of MFC (**a**), CMC (**b**) [33], F1 (**c**), F2 (**d**), F3 (**e**), F4 (**f**), F5 (**g**), F6 (**h**), F7 (**i**), F8 (**j**), and F9 (**k**) structures.

The laboratory-made MFC was obtained from a Eucalyptus globulus kraft pulp using a mechanical process followed by an environmentally friendly enzymatic treatment, resulting in a fiber suspension with 0.7 mm lengths and 22.3 µm widths (Table 2). This methodology resulted in a closed 3D network, with fibrils morphology changes at the microscale, as shown in Figure 3a. 

Additionally, after these treatments, 53.1% of fiber fraction remained in the sample, corresponding to 46.9% of fine elements. The coarseness values are related to the fiber population, fiber wall thickness, and fiber width, influencing fiber collapsibility and flexibility. The fiber deformations, such as kinks and curl, influence the strength properties since these parameters provide more open 3D network structures with low inter-fiber bonds. The low values of MFC indicated that strength properties can be increased, improving the inter-fiber bonds. Furthermore, these properties are also influenced by macrofibrils and fine elements that resulted from the fiber surface removal from the primary wall during fiber modification processes. In terms of drainability and water affinity, the MFC presented high °SR and WRV, respectively. These results showed that the effect of refining and enzymatic treatment enhanced more efficient fibrillation, which reduced drainability, increased water bond availability and accessibility, and consequently its retention capacity. These values are also due to the high parameters of intrinsic viscosity, related to DP, and C_COOH_ that result from degradation and intrinsic strength, and water interactions in a more local binding.

In our previous work, a micro/nanofibrillated cellulose (MFC/NFC) sample was investigated as an additive to produce low basis weight and high porosity materials [24]. This suspension presented 10% of particles in the nanoscale (less than 200 nm), being obtained by enzymatic treatments, refining, and homogenization processes applied to a Eucalyptus kraft pulp. The MFC sample used in the present study is at the microscale level, demonstrating different morphological, water interaction, and chemical properties compared to MFC/NFC. Higher values of fiber length and width, and consequently length/width ratio, were verified for MFC. These values are in agreement with the fiber fraction which is 63% higher compared to MFC/NFC. The low coarseness of MFC will promote fiber bonding, strength, and formation compared to MFC/NFC. MFC also showed a higher percentage of kinked and curled fibers than MFC/NFC, indicating that a more open structure with higher softness and lower strength can be produced. The low percentage of MFC macrofibrils and fine elements also confirms this report. The °SR values are in the same range and the WRV of MFC is lower due to the higher fiber modification treatment applied to the MFC/NFC. However, the intrinsic viscosity and C_COOH_ parameters showed higher values for the MFC sample than for the MFC/NFC sample. These differences depend on production methodology, energy consumption, enzyme type, and loading.

**Figure 4:**
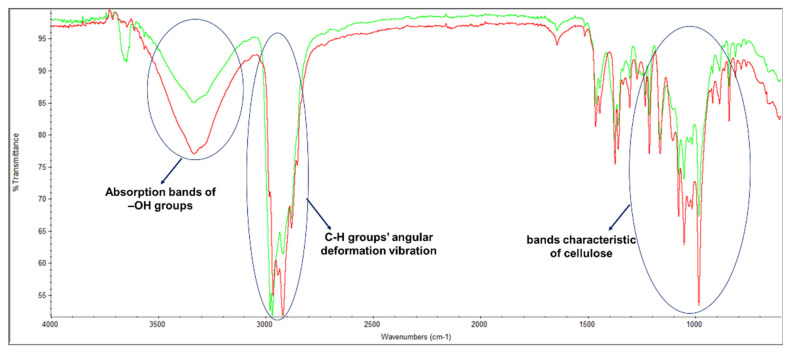
FTIR-ATR spectrum of a eucalyptus pulp (green) and MFC sample (red).

Additionally, the MFC chemical functional groups were identified using the FTIR-ATR method (Figure 4). *Eucalyptus globulus* kraft pulp was also analyzed since it is the starting point for obtaining MFC. Both samples were used to prepare laboratory-made structures and, consequently, to perform this analysis. Both FTIR spectrum showed the bands characteristic of cellulose [48,49]: angular deformations of C–H groups (~1374 cm^−1^), angular deformation of primary alcohols C–O bonds (~1164 cm^−1^), the absorption band of C–O–C bonds (~1015 cm^−1^), and β-glycosidic bonds between glucose units (~888 cm^−1^). The typical absorption bands of –OH groups at ~3336 cm^−1^ and C–H groups’ angular deformation vibration between 2880 and 2980 cm^−1^, approximately, were also verified. As dry laboratory-made structures were used in this analysis, the broadband at ~3336 cm^−1^ was not as visible, since the ability of the MFC sample to absorb more water can be demonstrated by the increase in the broad hydroxyl band. Despite this evidence, the main differences are found in these two bands, with the MFC sample presenting a higher band area and intensity. These results are in agreement with the chemical properties presented as well as the fiber modification treatments applied.

### 3.2. Characterization of CMC/EO Structure

The CMC described an interlaced, uniform, and open 3D network, as shown in Figure 3b. This matrix, due to its high porosity, can present the ability to hold large amounts of water and therapeutic molecules, and consequently, release them in a controlled manner. 

**Figure 5:**
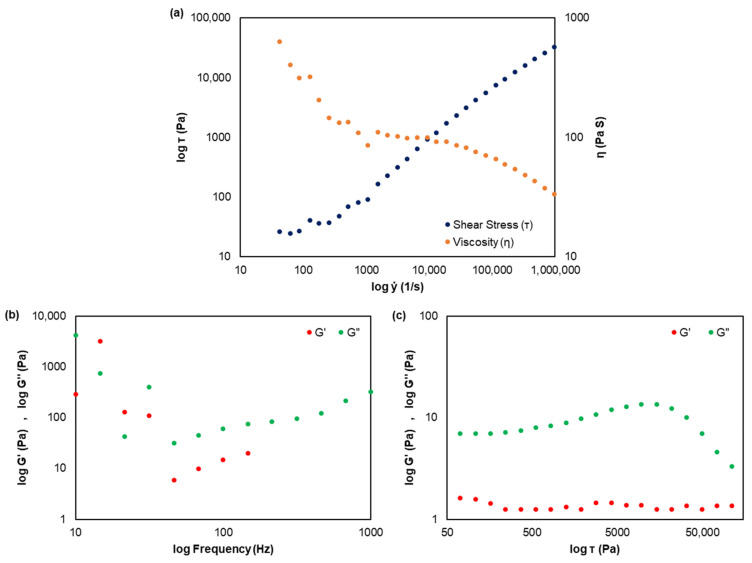
Rheological analysis of CMC/EO (5 mg/mL) at 23 ± 1 °C: shear stress and viscosity as a function of shear rate (**a**); storage and loss modulus as a function of frequency (**b**); and storage and loss modulus as a function of shear stress (**c**).

Although the CMC reagent has a high viscosity, this parameter depends on both concentration and temperature. As temperature increases, viscosity decreases, while higher concentration increases viscosity due to the intermolecular interactions between the CMC chains. Figure 5a presents the flow curve and viscosity as a function of the shear rate of the CMC/EO solution. The flow curve shape of this solution was similar to previous works, presenting almost a shear-thinning Newtonian behavior [50]. As this solution presents a low concentration (5 mg/mL—0.5% *w*/*v*), two shear-thinning behaviors are verified over two ranges of shear rates separated by a plateau value at a medium shear rate [51]. The change in rheological behavior corresponds to the inflection point in the sigmoid curve. Benchabane and Bekkour (2008) [52] also reported that from a concentration of 10 mg/mL (1% *w*/*v*), an initial shear-thickening behavior is observed, where the apparent viscosity increases with increasing shear rate, followed by a given shear rate by a shear-thinning behavior. This concentration is considered the overlap concentration since the rheological behaviors of low CMC concentration solutions are quite different from those of the higher concentrations [52]. As CMC is soluble in water up to 10 mg/mL, heat and agitation were required to prepare this solution, in a controlled pH range. These factors were optimized in order to produce a mixture that resembles a cream or lotion capable of retaining the essential oil and being effectively applied by spray on the surface structures. 

Oscillatory assays were also carried out on the CMC/EO sample (Figure 5b,c). Two different approaches were studied to record the corresponding modulus: (1) for a fixed strain, the oscillation frequency was changed (Figure 5b), and (2) at a fixed frequency (0.01 Hz), the shear stress was changed (Figure 5c) [43]. The results indicated that from the frequency of 0.1 Hz, the storage moduli could not be determined, as verified in Figure 5b. This evidence was also observed in the studies of [52]. Additionally, at the low concentration of CMC, the viscous properties are dominant compared to the elastic ones (G″ > G′) for a fixed frequency, as verified in Figure 5c. These results showed that CMC/EO presents more liquid behavior than gel behavior. The critical concentration of 1% (*w*/*v*) delimitates two different states of the CMC polymeric solution, a semi-diluted entangled 3D network, and a concentrated solution, in which viscoelastic properties appear in the polymeric solutions [52]. For this reason, a concentration of 0.5% was designed for the present work. The CMC/EO functions as a vehicle and chemical dispersant for eucalyptus essential oil, reducing the surface tension between oil and water, to be used in the development of biostructures for therapeutic molecule delivery. Obtaining durable essential oil fragrances on the structures’ surface is a challenge since these therapeutic molecules present high volatilization. The EO encapsulation in the 3D CMC matrix was a good route to control the fragrance release, with a duration between three and six weeks. These durability results were monitored over time, corresponding to air exposure and not when the material is stored and conditioned.

**Figure A1:**
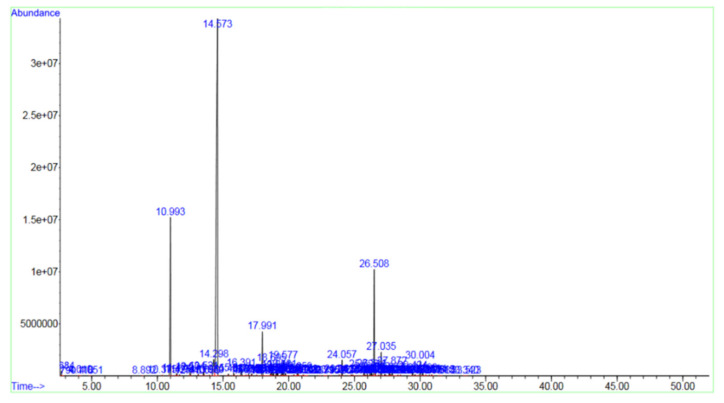
GC-MS chromatogram of the EO.

The study of the volatiles of EO revealed 105 different compounds, describing 99.95% of its chemical composition (Table 3). The GC-MS chromatogram obtained is presented in Figure A1 (Appendix A), in which the peaks presented correspond to the retention time of each compound in Table 3. Aromadendrene (7.72%), α-pinene (12.45%), and 1,8-cineole (59.19%) were the three major compounds, with the other components being present in trace amounts. Eucalyptol, or 1,8-cineole, is a monoterpenoid that is recognized for its anti-inflammatory, antimicrobial, antiseptic, antitussive, moisturizing, and revitalizing properties, and is indicated for relief of respiratory diseases, cough, bronchitis, cold, sinusitis, asthma (as a complementary measure), and use as a disinfectant, among others [53,54].

**Figure 6:**
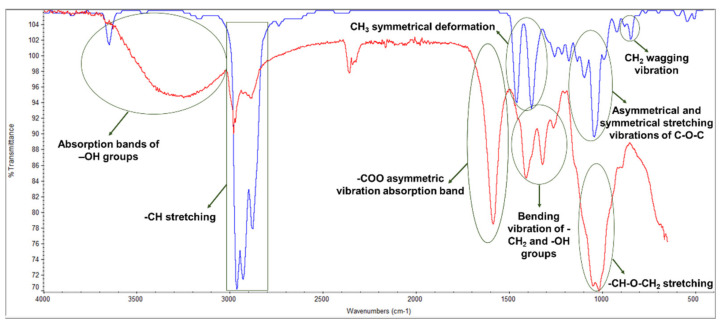
FTIR-ATR spectrum of CMC (red) and EO (blue).

Figure 6 presented the characteristic FTIR-ATR spectrum corresponding to the dried CMC sample and EO. The existence of molecular interactions between the 3D matrix of the retention agent and the therapeutic molecule results in different band displacements due to the formation or alteration of functional groups, which are evident with the concentration increase of essential oils [55,56]. The bands of CMC were detected at 3249 cm^−1^ referring to the stretching vibration of the –OH group, and at 2981 cm^−1^ attributed to the –CH stretching. The band at 1586.51 cm^−1^ refers to the –COO asymmetric vibration absorption peak. The intense band peaks at 1411 cm^−1^ and 1322 cm^−1^ refer to the bending vibration of –CH_2_ and –OH groups, respectively. The band around 1019 cm^−1^ is due to the –CH–O–CH_2_ stretching. Eucalyptus essential oil is a commercial product, and its composition is a mixture of different components, with the predominant terpenoid compound eucalyptol (1,8-cineole) [57]. Significant bands were observed at 3650 cm^−1^ corresponding to the –OH group; between 2880 and 2963 cm^−1^ assigned to methyl groups; at 1259 and 1043 cm^−1^ corresponding to the asymmetrical and symmetrical stretching vibrations of C–O–C, respectively; between 1379 and 1461 cm^−1^ attributed to the CH_3_ symmetrical deformation; and 921 cm^−1^ corresponding to CH_2_ wagging vibration [57,58]. The EO spectrum was also compared to the available libraries, presenting characteristic key bands to distinguish different components. The results of this analysis indicated that the eucalyptus essential oil used in the experiments showed correspondences with 1,8-cineole, α-pinene, and terpen-4-ol. These results are also in agreement with the GC-MS studies (Table 3 and Figure A1).

**Figure 7:**
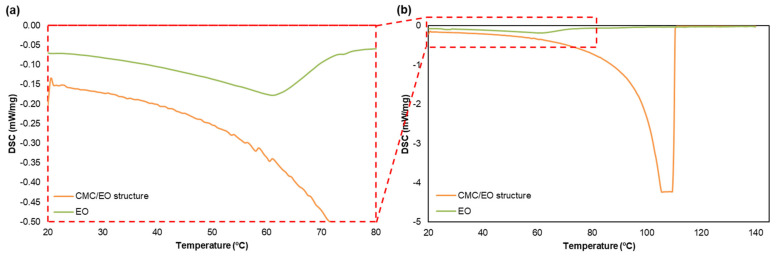
Comparison of DSC of the CMC/EO structure and the eucalyptus essential oil alone, (**a**) between 20 and 80 °C and (**b**) 20 and 140 °C.

The EO incorporation into the CMC structure enhances its retention and controlled release. CMC features a porous, interwoven, uniform, and open 3D network with the ability to retain therapeutic molecules such as essential oils. To incorporate the 3D CMC matrix into structures that serve as a delivery vehicle for localized applications, the EO release rate can be controlled by the properties of the carrier material, the properties of the active components, and also by external stimulants like temperature [59]. EO molecules can be loaded on the CMC stably for weeks, and then released with a temperature increase. The stability and volume of this carrier change with this increase. CMC damage can lead to a degradation-controlled release mechanism, while volume change usually leads to a swelling or shrinkage-controlled diffusion release mechanism [60]. This means that the controlled release of the temperature-activated CMC/EO works by trapping the therapeutic molecules on cooling and diffusing them on heating. The DSC technique has been used to quantify biomolecule release studies and the stability of complex structures such as delivery systems and pharmaceutical mixtures [33,61]. Thermal analysis methods were used to characterize, develop, and prove the therapeutic product quality. The DSC provided information about the CMC/EO performance and complex structure, characterizing its fusion behavior and release studies, and evaluated the evaporation process of the EO incorporated in this delivery system. The energy absorbed by the samples was determined by the temperature increase, which was important to characterize the EO release conditions and durability effect over the exposure time. The CMC/EO cooperativity can be accessed with this technique, considering a useful tool to design and develop delivery systems for different applications. The CMC/EO structure and EO were analyzed separately, understanding the EO retention process of this 3D matrix. Figure 7 showed the DSC spectrum of CMC/EO and the EO alone. This method was used in this study to evaluate the release process of EO molecules when incorporated into a CMC formulation. The release parameters were quantified by endothermic peaks, leading to a change in the baseline of the spectrum caused by the change in the sample’s mass throughout the assays [33]. The results indicated that EO alone is quite volatile, being fully vaporized in this temperature range, changing the phases from liquid to vapor. This result was also confirmed by the difference between the initial and final masses in the crucibles (0 g). The release of some components of the EO was also verified between 20 and 60 °C. The EO exposure to environmental conditions leads to the volatilization of its components, with potential negative effects on its stability for extended storage periods and, consequently, on its biological activities [62]. The time and amount of EO components in direct contact with the skin surface is low compared to when it is retained in a 3D matrix. Therefore, the EO incorporation in a system that releases it in a controlled way is essential for higher effectiveness of the prolonged therapeutic effect, as shown in the CMC/EO spectrum. The DSC curve for this sample indicated that EO release is retarded in a matrix of a functionalized cellulose, CMC. The temperature increase allowed the release and retention evaluation of EO and CMC molecules (20–70 °C). In the CMC structure, the majority of the essential oil molecules is incorporated in the 3D matrix. It was found that this encapsulation can prolong and improve the desired therapeutic effects. Additionally, the stability and integrity of the CMC/EO were further identified above 100 °C. Structural phase changes were not identified in this temperature range throughout the tests, and endothermic peaks were referred to the water removal during heating [33,61,63].

### 3.3. Characterization and Analysis of Formulations

The fibers used in the studied formulations were selected to be representative of hardwoods, like *Eucalyptus globulus* with a length of 0.8 mm, a width of 19.1 µm, coarseness of 6.6 mg/100 m, and fine content of 38%. The softwood fibers were mechanically treated to reach a 25 °SR, with a length of 1.8 mm, a width of 31.5 µm, coarseness of 15.5 mg/100 m, and fine content of 31%. Table 4 showed the morphological and water interaction properties of different formulations. The MorFi analyzer only can evaluate the parameters of fiber suspensions, so the presence of CMC/EO did not affect its fibrous composition. For this reason, the morphological properties of F3 and F5 were similar to F1, and F4 and F6 were similar to F2. Two distinct approaches were used to analyze these results: (1) investigate the effect of incorporating MFC in suspensions with a fixed percentage of hardwood and softwood pulps (F1 and F2); and (2) investigate the effect of the reduction and/or removal of softwood in formulations with MFC incorporation (F7, F8, and F9). By comparing formulations F1 and F2, the fiber length weighted by length, width, kinked fibers, and curl decreased with the incorporation of MFC. Inversely, the coarseness, rate of macrofibrils, and fines content were increased with the MFC incorporation. On the other hand, the softwood reduction and the hardwood increase in the formulations decreased all the morphological properties analyzed, when comparing F7 with F1. The results also indicated that the replacement of softwood by MFC fibers decreased all properties apart from the rate of macrofibrils and fines content when comparing F9 with F7. Formulation F8 presented morphological properties between F7 and F9.

Regarding the water interaction properties, all formulations presented °SR in the industrial range (23 to 34 °SR) used to produce high-quality products. In our previous study [24], it was found that the 10% MFC/NFC incorporation presented high °SR values, which can cause drainage difficulties, and consequently, runnability and efficiency issues. However, in this work, the addition of 10% MFC showed °SR values 29% lower compared to MFC/NFC. This result indicated that, in terms of drainability, MFC could be an innovative additive to produce added-value products up to the incorporation of 10%, which was not the case for MFC/NFC. Additionally, and overall, the MFC also increased the WRV properties, even with the softwood fibers decrease. WRV analysis was also carried out for formulations F3 (131.0 ± 1.2%) and F4 (223.0 ± 1.8%), and the results indicated that the addition of CMC/EO into bulk increases this property by 15% and 80% in the formulations without and with MFC, respectively.

**Figure 8:**
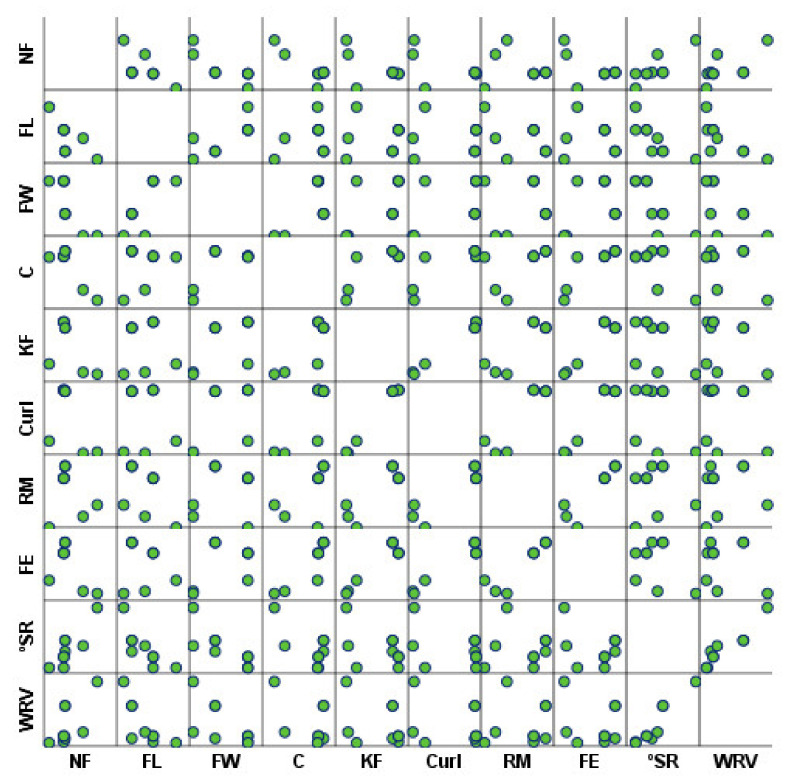
Correlation matrix of fiber morphology and water interaction properties.

The relationships between each fiber morphology and water interaction properties were also investigated using Pearson’s correlation matrix analysis (Table 5 and Figure 8). It was found that a total of 10 and 28 of the 100 pairwise correlations were considered significantly correlated at 0.05 and 0.01 levels, respectively. The direct and inverse relationship of each variable is determined by the positive or negative Pearson correlation coefficient, respectively. The *p*-value lower than 0.05 associated with this coefficient implies that the variables present a statistically significant association [64]. The interest relationships included the direct correlations between fiber width, coarseness, kinked fibers, curl, rate of macrofibrils, and fine elements, and inverse correlations between the number of fibers, fiber width, and coarseness. Furthermore, the °SR and WRV presented a direct association with the number of fibers and an inverse association with fiber length and width. Both parameters also showed a statistically significant positive association.

### 3.4. Characterization of Structures and Functional Properties Modeling

As mentioned before, new biodegradable systems applications require the use of sustainable materials such as cellulose fibers and derivatives, and natural products. The formulations were produced to investigate the effect of reducing and/or replacing reinforcing fibers with MFC as an additive, as well as the effect of applying a CMC/EO, with controlled retention and release properties, in the bulk and on the structure surfaces. The incorporation of polymeric CMC/EO formulations combined with a microscale natural fiber-based structures approach presents a potential strategy. The addition of cellulose microfibrils offers structural and mechanical properties, also contributing to the CMC/EO stabilization and the improvement of the functional properties of the structures functionalized with CMC and EO. The optimization of the structures and the crosslinking process involves a trade-off between achieving high absorbency and, at the same time, obtaining adequate mechanical strength and softness [62,65]. Figure 3 also presented the SEM images of the F1 to F9 structures. F1 showed a 3D network formed by two fibers with different morphologies: hardwood and softwood fibers (Figure 3c). The presence of fibrillation in the softwood fiber wall is a result of the applied refining process. The MFC incorporation, F2, contributed to the bonding between the hardwood and softwood fibers, filling the empty spaces and reducing the structure porosity (Figure 3d). The addition of CMC/EO in bulk, F3 and F4, promoted inter-fiber bonds with a more closed structure (Figure 3e,f). The CMC incorporation enhances intermolecular interactions with the surrounding environment due to the available hydroxyl groups, and consequently increases the water affinity, controlling the release of active molecules simultaneously, such as essential oils [32,33,66]. The application of the CMC/EO by the spray technique on the structure surface, F5 and F6, promoted a multilayer structure, with different separation levels (Figure 3g,h). This result is more visible in structures with MFC incorporated, synergistically promoting a more closed structure, occupying the empty spaces between fibers and fibrils. When the CMC/EO is introduced into the fiber suspension or applied to the cellulose structures, each 3D network depends on a different crosslinking mechanism, with the two networks remaining intensely entangled, and the eucalyptus essential oil retained in the 3D CMC matrix [65]. Additionally, the reduction of softwood fibers to 10%, F7, enhanced a more open and porous fibrous structure (Figure 3i). Contrary to the replacement by MFC, F9, a structure with reduced pore size and distribution is obtained (Figure 3k). From SEM images of the F8 multi-structured fibrous structures, it is possible to identify the different fiber fibrillation degrees, with inter-fiber bonding resulting from hardwood and MFC with a higher dosage (Figure 3j).

**Figure 9:**
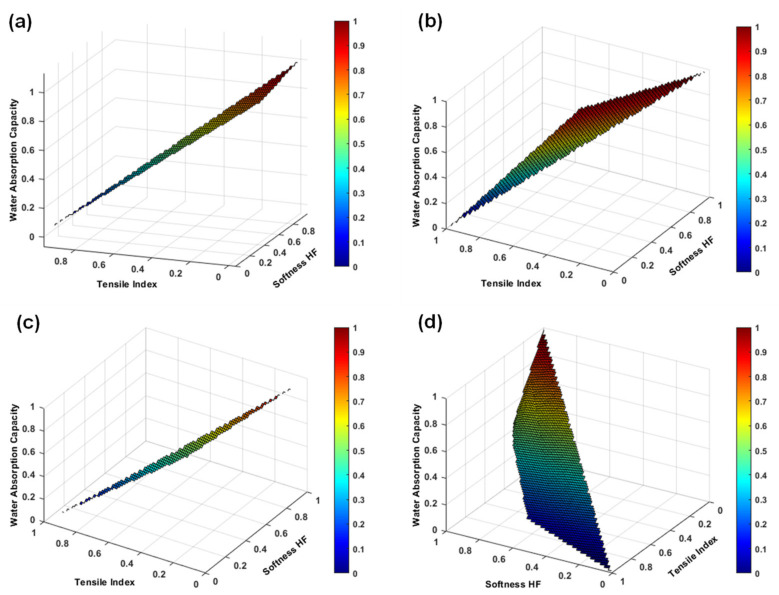
Evolution and prediction of softness HF, tensile index, and water absorption capacity from F1 and F2 (**a**), F3 and F4 (**b**), F5 and F6 (**c**), and F7 to F9 (**d**). The variables were normalized.

The effect of CMC/EO applied by two different techniques and MFC incorporation was evaluated through the functional properties. Figure 9 presented the evolution of the softness HF, tensile index, and water absorption capacity properties for the different formulations predicted using a 3D fiber modeling and structure simulation strategy. The analysis of this prediction was performed separately: (1) prediction of the effect of MFC addition in reference formulations, with fixed percentages of hardwood and softwood pulp fibers (Figure 9a); (2) prediction of the effect of applying CMC/EO in bulk (Figure 9b) and spray (Figure 9c), by changing the percentage of incorporated MFC; and (3) prediction of the effect of incorporating different percentages of softwood and MFC fibers, with a fixed percentage of hardwood pulp fibers (Figure 9d).

In a first approach, and analyzing Figure 9a, we considered a maximum MFC incorporation of 5% for the predictions, indicating that the normalized variable 0 corresponds to a formulation without this additive, and the normalized variable 1 corresponds to this maximum of incorporation. The computational results indicated that under these conditions the tensile index increased by 48% at the expense of reduced softness HF and water absorption capacity by 10% and 13%, respectively. Regarding the formulations produced, F1 presented a softness HF of 63.9, a tensile index of 13.4 Nm/g, and a water absorption capacity of 6.3 g/g, while F2 presented 62.8 of softness HF, 14.4 Nm/g of tensile index, and 6.0 g/g water absorption capacity. It was found that the MFC incorporation enhances the strength increase, without compromising the softness and absorption properties so characteristic of this type of material [23,24].

In a second approach, the addition of CMC/EO in bulk promoted a strength and absorption increase, and a softness reduction. Compared to its reference (F1), the tensile index and water absorption capacity of the F3 structures increased by 28% and 14%, respectively, at the expense of a 17% reduction in softness HF. On the other hand, compared to the reference with MFC (F2), the tensile index and the water absorption capacity of the F4 structures increased by 21% and 19%, respectively, at the expense of reduced softness HF by 19%. These results suggest that a CMC/EO presents a good potential to be introduced in new sustainable materials, aimed at high-value-added applications, such as facial masks, in which strength and absorption properties are essential [67]. Furthermore, it was predicted that MFC incorporation of up to 5% in formulations with CMC/EO also increased tensile index by 59% and decreased softness HF and water absorption capacity by 21% and 12%, respectively (Figure 9b).

When CMC/EO was applied by spraying on the structures’ surface, strength and absorption properties were also increased and softness was decreased compared to the references; however, this evolution was lower for strength and higher for softness and absorption, compared to formulations F3 and F4. Compared to reference F1, the tensile index and water absorption capacity of the F5 structures increased by 3% and 25%, respectively, at the expense of a 5% reduction in softness HF. In contrast, compared to the reference F2, the tensile index and the water absorption capacity of the F6 structures increased by 10% and 23%, respectively, at the expense of reduced softness HF by 8%. These results suggest that the application of a matrix with retention and controlled release properties on the surface of the structures achieves a trade-off between softness, strength, and absorption properties. This spraying technique can present a good potential to produce structures designed for dermal delivery applications of active substances more localized to the therapeutic target [37,38]. Moreover, it was predicted that MFC incorporation of up to 5% in formulations with CMC/EO applied on the surface also increased tensile index by 42% and decreased softness HF and water absorption capacity by 19% and 12%, respectively (Figure 9c).

The production of structures with high percentages of hardwood improves softness and absorption, so the strength properties must be balanced with fiber modification processes and/or the incorporation of reinforcing fibers or additives [24,67]. Formulations F7, F8, and F9 were investigated to understand this effect. A structure with 90% hardwood fibers and 10% softwood fibers (F7) showed values of 69.8 softness HF, 9.3 N/mm of tensile index, and 6.9 g/g of water absorption capacity. By replacing the reinforcing fibers in this formulation with 10% MFC (F9), the tensile index properties were increased by 38%, at the expense of reduced softness HF by 8% and water absorption capacity by 3%. The combination of these three cellulose sources (F8) also allowed to obtain a structure in different scales, with a trade-off between the functional properties: 68.1 of softness HF, 12.2 Nm/g of tensile index, and 6.7 of water absorption capacity. According to our previous study [24], the use of softwood reinforcing fibers with different origins, cooking and bleaching processes, and modification treatments, as well as additives only on the microscale presents a higher influence on the functional properties. When comparing structures with a fibrous composition similar to F7 in both studies, it was found that structures produced with softwood fibers with higher coarseness, and consequently, higher fiber wall thickness (present study), improved the softness by 3%, with a decrease in strength by 36% and absorption by 13%. Regarding the structures produced with the additives’ incorporation at the microscale (F9, in this study), the results indicated that the strength increased by 4%, with a decrease in softness by 11% and absorption by 8%, compared to structures produced with an additive both in micro and nanoscale (MFC/NFC). The production of structures with the conjugation of hardwood, softwood, and additive fibers was also carried out in both studies. Formulation F8 showed lower properties of softness (−4%), strength (−18%), and absorption (−11%) compared to a similar formulation in [24].

With this analysis, it was also possible to predict the influence of the amount of softwood fibers and MFC on the evolution of functional properties (Figure 9d). Two examples of these predictions can be highlighted: (1) structures produced with 90% hardwood fibers, 7% softwood fibers, and 3% MFC, and (2) structures produced with 90% hardwood fibers, 3% softwood fibers, and 7% MFC. The results of the first prediction indicated that structures with 69.1 of softness HF, 11.5 Nm/g of tensile index, and 6.8 g/g of water absorption capacity can be obtained. The structures produced with the second prediction showed softness HF of 65.1, tensile index of 12.4 Nm/g, and water absorption capacity of 6.7 g/g. These mathematical predictions can evaluate various scenarios in order to optimize the furnish process, and design and develop new materials, saving laboratory and industrial resources, in a quick and flexible response [44,47].

**Figure 10:**
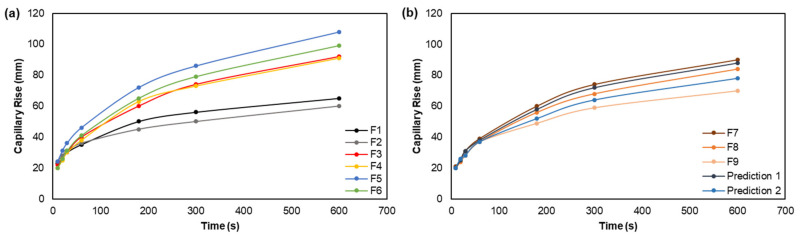
Evolution and prediction of capillary rise from F1 to F6 (**a**), and F7 to F9 (**b**).

Additionally, Figure 10 presented the evolution of the capillary rise properties for the different formulations. This property is another indicator of the absorption properties, which is in agreement with the water absorption capacity results. Capillary rise at 10 min decreased by 8% with the MFC addition, when comparing formulations F1 and F2. The addition of CMC/EO in bulk increased this property by 41% and 52% in structures without (F3 vs. F1) and with (F4 vs. F2) MFC incorporated. On the other hand, its addition to the structures’ surface promoted an increase in capillary rise by 66% in structures without MFC (F5 vs. F1) and 65% in structures with MFC (F6 vs. F2). The effect of combining the cellulose fibers with the CMC/EO in bulk can decrease permeability over time, whereby swelling of its particles tends to close the channels within the fibrous structure [65]. These results reflect the adhesion and cohesion forces of water molecules dependent on this property. The interactions of CMC functional groups enhanced the ascent of molecules through the fiber walls and between the pores [68]. The difference between the formulations is in the pore dimension and distribution promoted by the MFC, together with the CMC/EO.

Regarding the maximization of hardwood fibers in formulations (Figure 10b), the results indicated that the capillary rise decreased by 22% when comparing the F7 and F9 formulations, due to the 3D network with higher inter-fiber bonding caused by MFC. Formulation F8 also showed a capillary rise of 84 mm at 10 min. Furthermore, formulations F7, F8, and F9 presented lower capillary rises by 35%, 39%, and 57%, respectively, compared to similar formulations in [24]. The computational predictions also showed that 88 mm and 78 mm of capillary rise properties at 10 min can be obtained with prediction 1 (90% hardwood, 7% softwood fibers, and 3% MFC) and prediction 2 (90% hardwood, 3% softwood fibers, and 7% MFC), respectively.

The relationships between fiber morphology parameters and functional properties were also established using Pearson’s correlation matrix analysis (Table 6). Statistically significant positive correlations included the number of fibers for softness TS7, and coarseness, kinked fibers, curl, and fine elements for water absorption capacity. Statistically significant negative associations included fiber length weighted by length for softness TS7 and tensile index, and rate of macrofibrills and fine elements for the capillary rise.

The fiber length influences the strength properties, enhancing the several inter-fiber bonds. The inverse relationship obtained can be related to the short fibers caused by hardwood fibers and MFC fibers. As previously verified, inter-fiber bonds enhanced a more closed 3D network structure, improving tensile index and softness TS7 properties. Note that softness TS7 is related to the surface softness from the fibers and the structures, being inversely related to softness HF. Coarse fibers, with deformations, promote structures with higher bulk, and consequently higher water absorption capacity properties. The rate of macrofibrills and fine content, together with the fiber flexibility and collapsibility, are also other parameters that enhance inter-fiber bonding, and consequently, contribute to the decrease of capillary rise. The smaller pores in the 3D network structure caused by these interactions can restrict water progression by limited space for flow [68].

**Figure 11:**
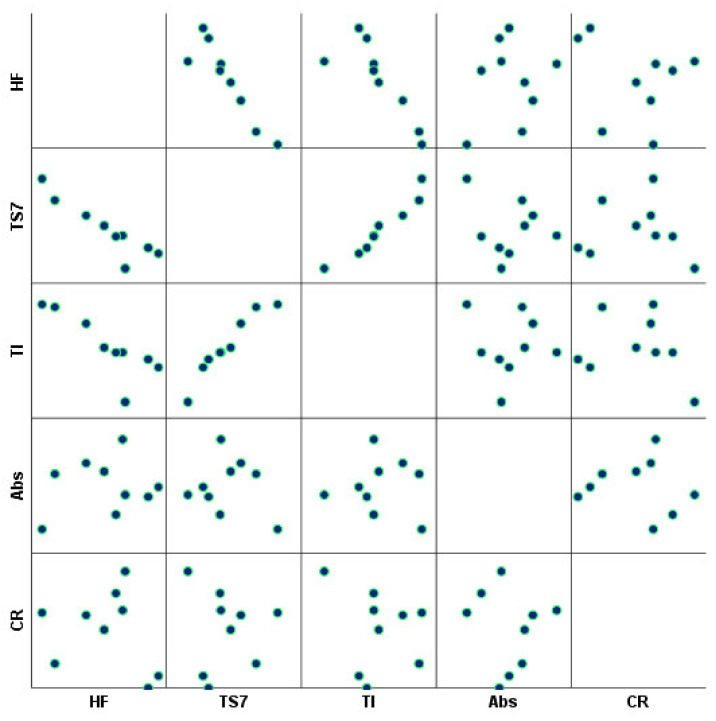
Correlation matrix of each functional property.

A correlation matrix detailing the relationship between each functional property was also assessed, as displayed in Figure 11. Statistically significant direct correlations included softness TS7 for tensile index (0.982, *p*-value < 0.01), while statistically significant inverse correlations included softness HF for softness TS7 (−0.913, *p*-value < 0.01) and tensile index (−0.827, *p*-value < 0.01). This result is expected since higher softness HF values are associated with lower softness TS7 values, and consequently, lower tensile index values [67]. It should also be noted that no statistically significant relationship was obtained between the water absorption capacity and capillary rise properties. This result indicates that several morphological, chemical and physical factors are contributing to the absorption properties, and, therefore, non-associated correlations were obtained with the softness and strength properties.

## 4. Conclusions

An experimental and computational methodology was implemented to design and engineer natural fiber-based biomaterials to replace the non-renewable fiber sources used to develop non-woven delivery systems. Cellulose-based structures were developed as the main support of delivery systems with MFC as an additive, which improved the strength at the expense of reduced softness and absorbency. Additionally, CMC matrix was incorporated into the system as a thermally stable vehicle to retain and control the release of the EO compounds. The application methods of CMC/EO were tested through bulk addition, and by spray application on the structures’ surfaces. Overall, the application of the CMC/EO led to an increase in tensile index and water absorption capacity values, regardless of the technique used. The absorption increase was more evident with spray application, while the strength increase was more pronounced with bulk addition. These results suggest that the spray technique can be used in structures aimed at a more localized therapy, like transdermal delivery systems, and the bulk addition method can be used in structures for different sustained applications, such as cosmetic facial masks or respiratory purposes. This optimization methodology highlights a concept to produce fibrous materials with innovative features for applications in the field of controlled and sustained release of a therapeutic or cosmetic active agent into the area to be treated.

## Figures and Tables

**Table 1:** Description of the formulations used in the studies.

Formulations	Eucalyptus Slush Pulp (%)	Softwood Pulp (%)	MFC (%)	CMC/EO (%)
F1	75	25	-	-
F2	75	25	2	-
F3	75	25	-	5
F4	75	25	2	5
F5 *	75	25	-	5
F6 *	75	25	2	5
F7	90	10	-	-
F8	90	5	5	-
F9	90	-	10	-

* In formulations F5 and F6, the CMC/EO was applied by spraying on the dry structures’ surface.

**Table 2:** Morphological, water interaction, and chemical properties of MFC sample.

Properties *	MFC (%) *
Morphological properties	
NF—Number of fibers (million/g)	24.7 ± 0.8
FL—Fiber Length weighted by Length (mm)	0.708 ± 0.004
FW—Fiber Width (µm)	22.3 ± 0.1
C—Coarseness (mg/100 m)	7.03 ± 0.01
KF—Kinked Fibers (%)	31.9 ± 0.8
Curl (%)	7.9 ± 0.0
RM—Rate of Macrofibrils (% in length)	1.182 ± 0.064
FE—Fine Elements (% in length)	46.9 ± 1.4
Water interaction properties	
°SR	93 ± 0
WRV (%)	481.9 ± 8.5
Chemical properties	
Viscosity (mL/g)	976.95 ± 0.05
DP	1713.95 ± 0.08
C_COOH_ (mmol/100 g)	11.3 ± 0.2

* Values reported are mean ± standard deviation.

**Table 3:** Chemical composition of EO assessed by GC analysis.

Compounds	Relative %	Retention Time (min)
β-Methylbutanal	0.16	2.683
Acetic Acid	0.01	2.790
1-Butanol	0.03	4.010
Isopropylacetone	0.01	4.109
Toluene	0.01	4.852
Isoamyl acetate	0.01	8.891
β-Pinene	0.04	10.377
**α-Pinene**	**12.45**	**10.993**
α-Fenchene	0.04	11.426
Camphene	0.16	11.474
2,4-Thujadiene	0.07	11.696
1,2,4,4-Tetramethylcyclopentene	0.01	12.439
β-Pinene	0.27	12.505
β-Myrcene	0.09	13.111
α-Phellandrene	0.33	13.521
cis-Anhydrolinalool oxide	0.02	13.651
α-Terpinene	0.05	13.957
p-Cymene	2.58	14.297
**1,8-Cineole**	**59.19**	**14.573**
γ-Terpinene	0.11	15.406
Nortricyclene	0.20	15.812
α -Dimethyl-1-Benzene	0.64	16.393
α-Ocimen	0.03	16.670
Linalool	0.07	16.751
γ-Methylene	0.01	16.877
Butanoic Acid	0.11	16.943
α-Fenchol	0.16	17.180
α-Campholenal	0.06	17.557
Isocitronellene	0.02	17.867
cis-Pinocarveol	3.08	17.993
Spiro[4.5]dec-6-ene	0.04	18.163
m-Mentha-4,8-diene	0.03	18.273
Trichloroacetic Acid	0.02	18.455
Methyl-1,4-heptadiene	0.03	18.543
Bicyclo[3.1.1]heptan-3-one	0.14	18.628
Bicyclo[2.2.1]heptan-3-one	0.77	18.695
Borneol	0.26	18.824
Bicyclo[3.1.1]heptan-3-one	0.37	19.049
Terpinen-4-ol	0.18	19.164
p-Cymen-8-ol	0.06	19.401
Thujol	0.43	19.482
α-Terpineol	0.97	19.578
2-Pinen-10-ol	0.25	19.748
α-Phellandrene Epoxide	0.03	19.899
2-Pinen-4-one	0.02	20.088
trans-Carveol	0.10	20.398
2-Oxabicyclo[2.2.2]octan-6-ol	0.02	20.513
trans-3-Pinen-2-ol	0.24	20.657
2-Methyladamantane	0.13	20.712
Myrtenyl Acetate	0.03	21.041
2-Cyclohexen-1-one	0.06	21.104
2-Cyclohexen-1-one	0.03	21.215
2,4-Octadienoic Acid	0.01	21.318
Piperitone	0.01	21.392
Borneol Acetate	0.03	22.338
2-Pinen-10-ol	0.02	22.715
p-Cymen-2-ol	0.02	22.797
β-Pinene	0.03	23.185
2-Oxabicyclo[2.2.2]octan-6-ol	0.07	23.853
α-Terpinyl Acetate	0.93	24.057
Isoledene	0.07	24.751
α-Copaene	0.06	24.829
Δ-Cadinene	0.06	24.892
α-Bourbonene	0.01	25.077
Junipene	0.03	25.383
Zingiberene	0.01	25.653
α-Gurjunene	0.42	25.727
Alloaromadendrene	0.07	25.849
β-Caryophyllene	0.13	25.978
β-Patchoulene	0.17	26.189
β-Gurjunene	0.46	26.311
Cyclohexene	0.03	26.377
**Aromadendrene**	**7.72**	**26.508**
α-Selinene	0.23	26.581
Pentalene	0.02	26.773
α-Humulene	0.07	26.843
α-Gurjunene	0.03	26.932
(-)-Alloaromadendrene	1.57	27.035
γ-Gurjunene	0.10	27.301
γ-Muurolene	0.07	27.390
β-Selinene	0.22	27.663
Ledene	0.11	27.722
Ledene	0.63	27.870
α-Muurolene	0.02	27.966
γ-Muurolene	0.01	28.073
β-Maaliene	0.02	28.144
α-Amorphene	0.05	28.317
Aromadendrene	0.03	28.461
l-Calamenene	0.05	28.517
β-Humulene	0.03	29.249
Epiglobulol	0.36	29.426
Palustrol	0.12	29.622
9-Norpresilphiperfolan-9-one	0.02	29.681
Spathulenol	0.04	29.843
(+)-Ledol	1.03	30.002
Veridiflorol	0.23	30.195
4′-Hydroxy-3′,5′-dimethylacetophenone	0.04	30.420
β-Eudesmene	0.04	30.450
Eremophila-1(10),11-diene	0.05	30.704
1H-Indene	0.08	30.886
β-Guaiene	0.02	30.982
β-Eudesmol	0.03	31.514
α-Eudesmol	0.02	31.584
Cyclododeca-4,8-dien-1-one	0.01	33.339
4-Ethyl-3-oxabicyclo[4.4.0]decane	0.01	33.524
***Total identified: 105 compounds***	***99.95%***	

**Table 4:** Morphological and water interaction of formulations studied.

Properties	F1	F2	F7	F8	F9
Morphological properties					
NF—Number of fibers (million/g)	20.0 ± 0.2	20.1 ± 0.2	19.1 ± 0.5	21.2 ± 0.1	22.0 ± 0.4
FL—Fiber Length weighted by Length (mm)	0.8 ± 0.0	0.7 ± 0.0	0.8 ± 0.0	0.8 ± 0.0	0.7 ± 0.0
FW—Fiber Width (µm)	19.6 ± 0.1	19.5 ± 0.1	19.6 ± 0.1	19.4 ± 0.1	19.4 ± 0.1
C—Coarseness (mg/100 m)	7.6 ± 0.1	7.7 ± 0.1	7.5 ± 0.2	6.9 ± 0.04	6.8 ± 0.1
KF—Kinked Fibers (%)	46.9 ± 0.5	45.8 ± 0.6	39.2 ± 0.6	37.7 ± 0.1	37.4 ± 0.6
Curl (%)	10.6 ± 0.1	10.5 ± 0.1	9.2 ± 0.1	8.8 ± 0.1	8.9 ± 0.1
RM—Rate of Macrofibrils (% in length)	0.610 ± 0.009	0.632 ± 0.010	0.520 ± 0.007	0.540 ± 0.019	0.561 ± 0.008
FE—Fine Elements (% in length)	42.0 ± 0.4	43.2 ± 0.3	37.7 ± 0.5	38.0 ± 0.2	39.1 ± 0.2
Water interaction properties					
°SR	23 ± 0	26 ± 0	23 ± 0	27 ± 0	34 ± 0
WRV (%)	113.2 ± 0.1	123.4 ± 1.5	100.1 ± 1.0	142.8 ± 2.7	296.6 ± 7.2

Note: Values reported are mean ± standard deviation.

**Table 5:** Pearson’s correlation values for each fiber morphology and water interaction properties.

		NF	FL	FW	C	KF	Curl	RM	FE	°SR	WRV
**NF**	Pearson’s correlation	1	−0.663	−0.790 *	−0.887 **	−0.544	−0.532	−0.198	−0.562	0.845 **	0.669 *
	*p*-value		0.052	0.011	0.001	0.130	0.140	0.610	0.115	0.004	0.049
**FL**	Pearson’s correlation	−0.663	1	0.762 *	0.279	−0.018	−0.083	−0.484	−0.162	−0.807 **	−.0755 *
	*p*-value	0.052		0.017	0.467	0.963	0.831	0.187	0.677	0.009	0.019
**FW**	Pearson’s correlation	−0.790 *	0.762 *	1	0.660	0.567	0.513	0.131	0.390	−0.805 **	−0.668 *
	*p*-value	0.011	0.017		0.053	0.111	0.158	0.738	0.299	0.009	0.049
**C**	Pearson’s correlation	−0.887 **	0.279	0.660	1	0.829 **	0.840 **	0.625	0.879 **	−0.662	−0.472
	*p*-value	0.001	0.467	0.053		0.006	0.005	0.072	0.002	0.052	0.200
**KF**	Pearson’s correlation	−0.544	−0.018	0.567	0.829 **	1	0.995 **	0.881 **	0.944 **	−0.447	−0.310
	*p*-value	0.130	0.963	0.111	0.006		0.000	0.002	0.000	0.228	0.418
**Curl**	Pearson’s correlation	−0.532	−0.083	0.513	0.840 **	0.995 **	1	0.912 **	0.969 **	−0.395	−0.252
	*p*-value	0.140	0.831	0.158	0.005	0.000		0.001	0.000	0.292	0.513
**RM**	Pearson’s correlation	−0.198	−0.484	0.131	0.625	0.881 **	0.912 **	1	0.920 **	−0.021	0.082
	*p*-value	0.610	0.187	0.738	0.072	0.002	0.001		0.000	0.956	0.835
**FE**	Pearson’s correlation	−0.562	−0.162	0.390	0.879 **	0.944 **	0.969 **	0.920 **	1	−0.345	−0.191
	*p*-value	0.115	0.677	0.299	0.002	0.000	0.000	0.000		0.363	0.623
**°SR**	Pearson’s correlation	0.845 **	−0.807 **	−0.805 **	−0.662	−0.447	−0.395	−0.021	−0.345	1	0.936 **
	*p*-value	0.004	0.009	0.009	0.052	0.228	0.292	0.956	0.363		0.000
**WRV**	Pearson’s correlation	0.669 *	−0.755 *	−0.668 *	−0.472	−0.310	−0.252	0.082	−0.191	0.936**	1
	*p*-value	0.049	0.019	0.049	0.200	0.418	0.513	0.835	0.623	0.000	

* *p*-value < 0.05; ** *p*-value < 0.01.

**Table 6:** Pearson’s correlation values for fiber morphology and functional properties.

		HF	TS7	TI	ABS	CR
**NF**	Pearson’s correlation	−0.526	0.704 *	0.634	−0.620	0.067
	*p*-value	0.146	0.034	0.067	0.075	0.864
**FL**	Pearson’s correlation	0.562	−0.782 *	−0.868 **	0.232	0.440
	*p*-value	0.115	0.013	0.002	0.548	0.235
**FW**	Pearson’s correlation	0.539	−0.601	−0.612	0.631	−0.028
	*p*-value	0.135	0.087	0.080	0.069	0.942
**C**	Pearson’s correlation	0.404	−0.445	−0.293	0.754 *	−0.411
	*p*-value	0.281	0.231	0.445	0.019	0.271
**KF**	Pearson’s correlation	0.314	−0.147	0.033	0.799 **	−0.638
	*p*-value	0.411	0.707	0.932	0.010	0.065
**Curl**	Pearson’s correlation	0.268	−0.105	0.082	0.782 *	−0.664
	*p*-value	0.486	0.789	0.834	0.013	0.051
**RM**	Pearson’s correlation	0.010	0.223	0.426	0.592	−0.764 *
	*p*-value	0.979	0.564	0.253	0.093	0.017
**FE**	Pearson’s correlation	0.211	−0.092	0.113	0.732 *	−0.670 *
	*p*-value	0.585	0.814	0.772	0.025	0.048

* *p*-value < 0.05; ** *p*-value < 0.01. HF: softness HF; TS7: softness TS7; TI: tensile index; ABS: water absorption capacity; CR: capillary rise.

## Data Availability

The raw/processed data required to reproduce the above findings cannot be shared at this time as the data also form part of an ongoing study.
